# Epicardial fat thickness predicts severe coronary artery disease and high mortality risk among ST-elevation myocardial infarction patients

**DOI:** 10.1186/s44156-025-00087-y

**Published:** 2025-07-21

**Authors:** Heba M. El-Naggar, Jacqueline G. Abdel-Maseh, Hosam Hasan-Ali, Shimaa S. Khidr

**Affiliations:** Department of Cardiovascular Medicine, Assiut University Heart Hospital, Assiut, 71526 Egypt

**Keywords:** Echocardiography, Epicardial fat thickness, ST-elevation myocardial infarction, Syntax score, Grace score

## Abstract

**Background:**

Epicardial adipose tissue has been identified as a significant marker in the assessment of coronary artery disease (CAD), with a possible impact on the development of acute coronary events including ST-elevation myocardial infarction (STEMI).

**Aim:**

To assess the association and predictability of echocardiographic-measured epicardial fat thickness (EFT) for the severity of CAD and mortality risk among STEMI patients.

**Methods:**

This study included 159 STEMI patients who underwent primary percutaneous coronary intervention (PPCI) and survived the in-hospital duration. Anthropometric measurements, lipid profiles, and angiographic data were recorded. The correlations between echo-measured EFT and CAD severity indicated by the syntax score (SS) were assessed. In-hospital and 6-month major adverse cardiovascular events (MACE) were reported, and mortality risk was evaluated using the Grace score.

**Results:**

Among the study population, 104 patients (65.4%) had low SS, 45 patients (28.3%) had moderate SS, and 10 patients (6.3%) had high SS. STEMI patients with moderate/high SS had significantly larger EFT. EFT showed a significant correlation with BMI (r = 0.57), fat mass (kg) (r = 0.44), LDL (r = 0.40), the syntax score (r = 0.74), and the Grace score (r = 0.68), (*p* < 0.001 for all). Our ROC-derived cutoff value of EFT ≥ 5.45 mm significantly discriminated STEMI patients with moderate/high-SS, high coronary thrombus burden, 6-months high mortality risk, and 6-months MACE with reasonable respective sensitivity and specificity. Increased EFT independently predicted moderate/high-SS and high mortality risk on multivariable regression analysis.

**Conclusion:**

Echo-measured EFT ≥ 5.45 mm might be a reliable non-invasive marker for predicting CAD severity, high coronary thrombus burden, 6-months high mortality risk, and 6-months MACE among STEMI patients.

**Supplementary Information:**

The online version contains supplementary material available at 10.1186/s44156-025-00087-y.

## Key Points

- Echo-derived EFT is a reliable and versatile tool for risk assessment among STEMI patients.
- STEMI patients with moderate/high SS had significantly larger EFT and PFT.
- EFT ≥ 5.45 mm significantly discriminated STEMI patients with moderate/high-SS.
- EFT ≥ 5.45 mm predicted high thrombus burden, 6-months high mortality risk, and 6-months MACE.
- EFT might be incorporated in risk models for 1ry and 2ry prevention of CAD.

**Supplementary Information:**

The online version contains supplementary material available at 10.1186/s44156-025-00087-y.

## Introduction

Epicardial adipose tissue (EAT) is the visceral fat located between the myocardium and the visceral pericardium. EAT-induced inflammation has been implicated in the pathogenesis of coronary artery disease (CAD) [1].

EAT expresses several cytokines involved in vascular plaque formation. Excess release of inflammatory cytokines, endothelial dysfunction, and increased oxidative stress all share in the development and progression of coronary atherosclerosis [2]. Moreover, it has been shown that EAT influences plaque instability via its paracrine effect on nearby coronaries and hence impacts the development of acute coronary syndromes (ACS) [3].

Echocardiography has been identified as a reliable imaging tool for quantifying epicardial fat thickness (EFT) [4]. This study provided a comprehensive echocardiographic assessment of EFT and aimed primarily to evaluate its association with the severity of CAD assessed by syntax score (SS) among ST-elevation myocardial infarction (STEMI) patients who underwent primary percutaneous coronary intervention (PPCI). As a secondary aim, we assessed the association of EFT and possible anthropometric determinants of atherosclerosis (BMI, fat mass), coronary thrombus burden, post-PCI no-reflow, mortality risk assessed by Grace score, and 6-month adverse events. 

## Methods

This was a cross-sectional observational study conducted between January 2022 and March 2024. The study included 159 STEMI patients who underwent PPCI and survived the in-hospital duration. Patients with any of the following were excluded: previous coronary artery bypass grafting, advanced kidney or liver disease, malignancy, pericardial disease, familial hypercholesterolemia, cardiogenic shock, poor echocardiographic window, and those who did not consent to the study protocol (Figure S1).

Index STEMI (anterior and non-anterior), total ischemic time (TIT), and 90 min ST-segment resolution were recorded. Anthropometric data included: weight, height, body surface area (BSA), body mass index (BMI), waist and hip circumferences, and waist/hip ratio. Fat mass was calculated in (%) and (kg) according to the Navy method [5]. Peak creatine-kinase (CK), total cholesterol, triglycerides, low-density lipoprotein (LDL), and high-density lipoprotein (HDL) were assessed. Atherogenic Index of Plasma (AIP) was calculated as the logarithm of triglycerides to HDL ratio [6].

Angiographic and procedural data included assessment of the coronary thrombus grade (grades 4 and 5 denoted high thrombus burden) [7] and post-PCI no-reflow (TIMI-flow ≤ 2). The severity of CAD was evaluated using the syntax score (SS) [8], accordingly, patients were classified as low-SS (≤ 22), intermediate-SS (> 22–32), and high-SS (> 32). 

Echocardiography was performed pre-hospital discharge using (GE/Vivid-S6) ultrasound machine. Both epicardial (EFT) and pericardial (PFT) fat thickness were measured in parasternal long-axis (PLAX) and short-axis (PSAX) views at end-systole (ES) and end-diastole (ED) over three cardiac cycles, and averaged values were recorded for each. With enhanced depth setting, EFT was measured as the largest perpendicular distance to the RV free wall along the midline of the ultrasound beam, with reference to the aortic annular plane in PLAX and the interventricular septum at the papillary muscle level in PSAX. PFT was measured as the space just outside the epicardial fat (Figure S2) [9].

Cardiac magnetic resonance (CMR) was performed for a subset of 43 patients within 30 days from the incident MI using 1.5-Tesla scanner (Philips/Ingenia/Release: 4.1.3.0). Standard steady-state-free-precession (SSFP) images of the left (LV) and right (RV) ventricles in the horizontal and vertical long-axis, and LV outflow tract views, and a stack of short-axis images for volumetric and functional assessment were acquired. Using dedicated software (MR-Workspace: R2.6.3.1), manual tracing on the 4-chamber (horizontal long-axis) view at ED was performed for maximal epicardial (EFA) and pericardial (PFA) fat areas (Figure S2).

In-hospital major adverse cardiovascular events (MACE) (re-infarction, heart failure, and/or serious arrhythmias) were reported. Mortality risk was assessed using the Grace score [10] defining low, intermediate, and high in-hospital mortality risk categories as: (49–125), (126–154), and (155–319), respectively, and those at 6 months as: (27–99), (100–127), and (128–263), respectively. Follow-up 6-months MACE was defined as the composite of acute myocardial infarction whether STEMI or Non-STEMI, target lesion revascularization, heart failure, stroke, and death.

The study was approved by our institutional ethical committee (IRB: 17100627). Patients provided informed consent for participation.

### Statistical analysis

SPSS (version 25.0) was used. Continuous variables were presented as mean ± standard deviations or median (interquartile range) and were compared using an independent sample t-test and Mann–Whitney-U test, respectively. One-way ANOVA was used to compare SS-subgroups. Categorical variables were described as numbers (percentage) and were compared by Chi^2^-test. Pearson correlation was used for continuous variables. Receiver operating characteristic (ROC) analysis was used to determine the cutoff of EFT for predicting moderate/high-SS, high thrombus burden, high mortality risk, and 6-month MACE. Kaplan–Meier survival analysis for the outcome of cumulative MACE-free survival at 6-month was performed to compare patient groups categorized according to the generated EFT cutoff. Logistic and Linear regression analyses were used to test the predictability of EFT for different dependent variables. Univariable and multivariable logistic regression analyses were performed for possible predictors of severe CAD (moderate/high SS) and Grace score-derived 6-month high mortality risk. Variables with p < 0.05 on univariable regression were included in multivariable models. Reliability analysis for intra- and inter-observer variability was performed for echo-measured epi- and pericardial fat thickness and CMR-measured areas using intra-class correlation coefficient. p < 0.05 indicated statistical significance.

## Results

This study included 159 STEMI patients who underwent PPCI, of whom 65.4% had low SS, 28.3% had moderate SS, and only 6.3% had high SS (Figure S1). Moderate and high-SS patients were grouped (moderate/high-SS group) and studied with respect to the low-SS ones (Table 1). The moderate/high-SS group was significantly older and had significantly larger waist and hip circumferences, BSA, BMI, and fat mass, and significantly higher total cholesterol, LDL, and triglycerides and hence higher AIP. Both groups had comparable extent of the index STEMI, TIT, peak-CK, 90-min ST-resolution, and comparable in-hospital MACE. The moderate/high-SS group had a significantly higher Grace score. Six-month follow-up data (n = 148) showed that 16 patients (10.8%) had MACE, with significantly higher rate among the moderate/high-SS group compared to low-SS group (25.5% vs 3.1%, *p* < 0.001), most of whom had heart failure (15.7% vs 2.1%), myocardial infarction (7.9% vs 1.0%), stroke (2.0% vs 0.0%), (*p* = 0.001), yet there were no deaths among both groups at 6 months (Figure S1).

**Table 1:** Clinical data of the study population

	All individuals (n = 159)	Low SS (n = 104) (65.4%)	Moderate/High SS (n = 55) (34.6%)	*P-*value
Age (years)	56.10 ± 11.95	54.69 ± 12.74	58.76 ± 9.86	0.04
Male gender	137 (86.2%)	90 (86.5%)	47 (85.5%)	0.85
Smoking	101 (63.5%)	52 (50.0%)	49 (89.1%)	< 0.001
Hypertension	37 (23.3%)	20 (19.2%)	17 (30.9%)	0.09
Diabetes mellitus	43 (27.0%)	27 (26.0%)	16 (29.1%)	0.67
Previous ACS	20 (12.6%)	13 (12.5%)	7 (12.7%)	0.96
Waist circumference (cm)	127.00 ± 15.34	122.51 ± 15.39	135.49 ± 11.15	< 0.001
Hip circumference (cm)	124.54 ± 14.78	120.35 ± 14.97	132.47 ± 10.66	< 0.001
Waist/Hip ratio	1.01 ± 0.02	1.01 ± 0.01	1.02 ± 0.02	0.10
BSA (m^2^)	1.86 ± 0.14	1.82 ± 0.13	1.92 ± 0.14	< 0.001
BMI (kg/m^2^)	28.13 ± 4.31	26.23 ± 2.90	31.71 ± 4.30	< 0.001
Fat mass (kg)	31.63 ± 12.09	27.81 ± 10.14	38.86 ± 12.25	< 0.001
Fat mass% (Navy)	39.34 ± 11.25	36.49 ± 10.65	44.73 ± 10.43	< 0.001
Lipid profile				
Total cholesterol (mg/dl)	222.08 ± 60.04	205.88 ± 50.06	250.42 ± 65.78	< 0.001
LDL (mg/dl)	140.00 ± 50.16	125.45 ± 41.67	165.47 ± 53.88	< 0.001
HDL (mg/dl)	40.10 ± 8.86	41.13 ± 8.99	38.28 ± 8.40	0.06
Triglycerides (mg/dl)	173.50 ± 89.9	154.34 ± 75.91	207.04 ± 102.64	0.001
Atherogenic plasma index	0.59 ± 0.24	0.54 ± 0.22	0.69 ± 0.24	< 0.001
Anterior STEMI	113 (71.1%)	73 (70.2%)	40 (72.7%)	0.73
Total ischemic time (hrs)	5.04 ± 1.86	5.16 ± 1.93	4.82 ± 1.71	0.26
Peak-CK	1436 (900–2358)	1302 (894–2087)	1587 (1117–2596)	0.06
Complete ST-resolution	149 (93.7%)	99 (95.2%)	50 (90.9%)	0.57
Grace score	102.19 ± 26.96	87.43 ± 15.37	130.11 ± 21.54	< 0.001
Grace in-hospital risk				
Low (49–125)	134 (84.3%)	104 (100%)	30 (54.5%)	< 0.001
Intermediate (126–154)	18 (11.3%)	0 (0.0%)	18 (32.7%)	
High (155–319)	7 (4.4%)	0 (0.0%)	7 (12.7%)	
Grace 6-months risk				
Low (27–99)	75 (47.2%)	74 (71.2%)	1 (1.8%)	< 0.001
Intermediate (100–127)	63 (39.6%)	30 (28.8%)	33 (60.0%)	
High (128–263)	21 (13.2%)	0 (0.0%)	21 (38.2%)	
In-hospital MACE	17 (10.7%)	9 (8.7%)	8 (14.5%)	0.25
Reinfarction	1 (0.6%)	0 (0.0%)	1 (1.8%)	0.45
Heart failure	7 (4.4%)	4 (3.8%)	3 (5.5%)	
Arrhythmias	9 (5.7%)	5 (4.8%)	4 (7.3%)	
6-months MACE (n = 148)	16 (10.8%)	3 (3.1%)	13 (25.5%)	< 0.001

*ACS* acute coronary syndrome, *BMI* body mass index, *BSA* body surface area, *CK* creatine-kinase, *HDL* high density lipoprotein, *LDL* low density lipoprotein, *MACE* major adverse cardiovascular events, *SS* syntax score, *STEMI* ST-elevation myocardial infarction

### Echocardiographic, CMR, and angiographic data among the study groups

**Fig. 1:**
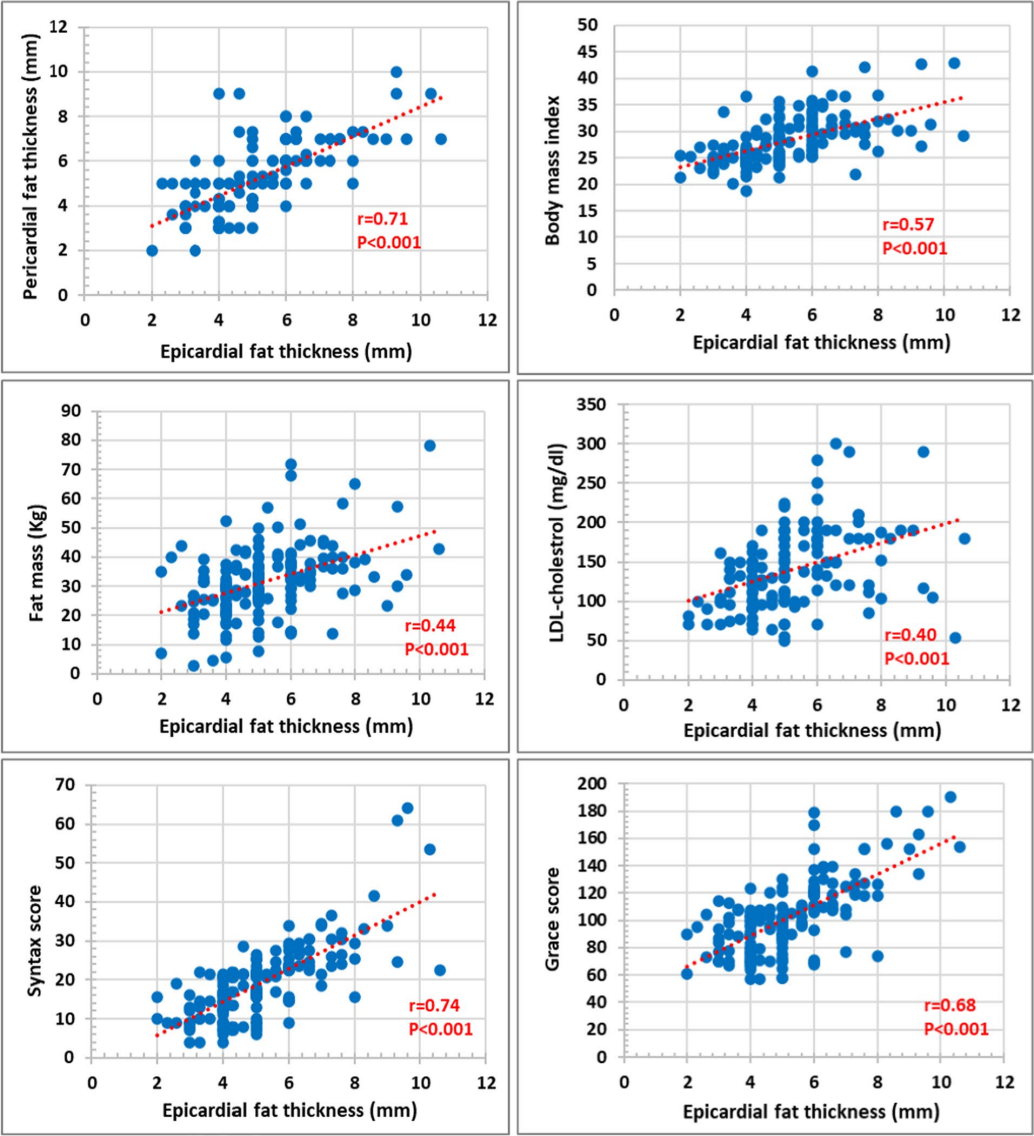
Correlations between epicardial fat thickness and each of pericardial fat thickness (parasternal long-axis at end-systole) and body mass index (upper panel), fat mass (Kg) and LDL-cholesterol (middle panel), Syntax score and Grace score (lower panel). *r* (correlation coefficient) and *p* (significance of correlation)

Echocardiographic data (Table 2) showed comparable LV dimensions and function among both groups, however, the moderate/high-SS had significantly larger EFT and PFT. Noticeably, this was consistent for all measured EFT and PFT from either PLAX and SAX views, and in either ES or ED frames. EFT showed significant correlation with corresponding PFT (r = 0.71), BMI (r = 0.57), fat mass (kg) (r = 0.44), LDL (r = 0.40), syntax score (r = 0.74), and Grace score (r = 0.68), (*p* < 0.001 for all) (Fig. 1).

**Table 2:** Echocardiographic data of the study population

	All individuals (n = 159)	Low SS (n = 104) (65.4%)	Moderate/High SS (n = 55) (34.6%)	*P-*value
Left ventricular dimensions and function				
LV EDD (cm)	5.09 ± 0.46	5.10 ± 0.46	5.08 ± 0.45	0.74
LV ESD (cm)	3.94 ± 0.44	3.92 ± 0.45	3.99 ± 0.42	0.36
LV EF (%)	47.06 ± 8.63	47.85 ± 8.89	45.56 ± 7.98	0.11
Epicardial fat thickness (mm)				
EFT-ES PLAX (n = 159)	5.18 ± 1.62	4.34 ± 1.01	6.76 ± 1.36	< 0.001
EFT-ED PLAX (n = 159)	4.17 ± 1.59	3.32 ± 0.94	5.78 ± 1.34	< 0.001
EFT-ES PSAX (n = 147)	5.23 ± 1.59	4.44 ± 0.95	6.88 ± 1.35	< 0.001
EFT-ED PSAX (n = 147)	4.15 ± 1.55	3.36 ± 0.92	5.78 ± 1.29	< 0.001
Pericardial fat thickness (mm)				
PFT-ES PLAX (n = 142)	5.24 ± 1.55	4.48 ± 1.17	6.64 ± 1.14	< 0.001
PFT-ED PLAX (n = 141)	4.71 ± 1.68	3.81 ± 1.15	6.34 ± 1.22	< 0.001
PFT-ES PSAX (n = 115)	5.21 ± 1.46	4.48 ± 1.09	6.54 ± 1.05	< 0.001
PFT-ED PSAX (n = 115)	4.75 ± 1.61	3.86 ± 1.06	6.34 ± 1.14	< 0.001

*ED* end-diastole, *EDD* end-diastolic dimension, *EF* ejection fraction, *EFT* epicardial fat thickness, *ES* end-systole, *ESD* end-systolic dimension, *LV* left ventricle, *PFT* pericardial fat thickness, *PLAX* parasternal long-axis, *PSAX* parasternal short-axis, *SS* syntax score

For patients who underwent CMR (n = 43) (Table S1), our results showed comparable LV and RV volumes and function, and EFA among both groups, but with significantly larger PFA among the moderate/high-SS group. Results showed a significant moderate correlation between echo-measured EFT and PFT and corresponding CMR-measured areas (Table S2). This significant moderate correlation was consistently noted for echo-measured EFT and PFT on either parasternal long- or short-axis view at both end-systolic or end-diastolic frames in relation to CMR-derived diastolic areas.

Reliability analysis for intra- and inter-observer variability was performed among a random sample of 42 patients for echo-measured EFT and PFT, and among 13 patients for corresponding CMR-measured areas. Intra-class correlation coefficients were significantly high for intra- and inter-observer variability of EFT (0.92 and 0.90, respectively) and PFT (0.92 and 0.88, respectively), (p < 0.001 for all). Inter-observer coefficients for CMR-EFA and PFA were 0.97 and 0.98, respectively, (p < 0.001 for both).

Regarding the angiographic data (Table 3), the moderate/high-SS group had significantly higher rates of aorto-ostial lesions, LM and three-vessel disease, and proximal culprit vessel affection compared to the low-SS group. Moreover, they had significantly higher rates of pre-PCI TIMI-flow grade-0 and high thrombus burden, as well as higher rates of no-reflow, and hence higher rates of using GP IIb/IIIa inhibitors. A comparative analysis of the three SS categories was performed (Table S3).

**Table 3:** Angiographic data of the study population

	All individuals (n = 159)	Low SS (n = 104) (65.4%)	Moderate/High SS (n = 55) (34.6%)	*P-*value
Aorto-ostial lesions	4 (2.5%)	0 (0.0%)	4 (7.3%)	0.005
Left-main disease	8 (5.0%)	0 (0.0%)	8 14.5%)	< 0.001
Diseased vessels				
Single-vessel	69 (43.4%)	65 (62.5%)	4 (7.3%)	< 0.001
Two-vessel	63 (39.6%)	31 (29.8%)	32 (58.2%)	
Three-vessel	27 (17.0%)	8 (7.7%)	19 (34.5%)	
Num. lesions/patient	2.02 ± 1.08	1.63 ± 0.86	2.76 ± 1.07	< 0.001
Culprit-vessel				
LAD	117 (73.6%)	76 (73.1%)	41 (74.5%)	0.22
LCX	11 (6.9%)	5 (4.8%)	6 (10.9%)	
RCA	31 (19.5%)	23 (22.1%)	8 (14.5%)	
Proximal lesions	90 (56.6%)	50 (48.1%)	40 (72.7%)	0.003
Pre-PCI TIMI-flow				
TIMI-0	121 (76.1%)	71 (68.3%)	50 (90.9%)	0.01
TIMI-1	32 (20.1%)	28 (26.9%)	4 (7.3%)	
TIMI-2	3 (1.9%)	3 (2.9%)	0 (0.0%)	
TIMI-3	3 (1.9%)	2 (1.9%)	1 (1.8%)	
Thrombus burden				
Low-burden (grade 1–3)	73 (45.9%)	69 (66.3%)	4 (7.3%)	< 0.001
High-burden (grade 4–5)	86 (54.1%)	35 (33.7%)	51 (92.7%)	
Post-PCI TIMI-flow				
TIMI-0	1 (0.6%)	0 (0.0%)	1 (1.8%)	0.007
TIMI-1	2 (1.3%)	0 (0.0%)	2 (3.6%)	
TIMI-2	10 (6.3%)	3 (2.9%)	7 (12.7%)	
TIMI-3	146 (98.1%)	101 (97.1%)	45 (81.8%)	
No-reflow (TIMI-flow ≤ 2)	13 (8.2%)	3 (2.9%)	10 (18.2%)	0.001
GP-IIb/IIIa inhibitors	36 (22.6%)	13 (12.5%)	23 (41.8%)	< 0.001
Syntax score	19.44 ± 9.43 20.5 (13.0–23.0)	14.55 ± 5.24 15.5 (9.0–19.3)	28.69 ± 8.65 28.6 (23.5–30.5)	< 0.001 < 0.001

*LAD* left anterior descending, *LCX* left circumflex, *GP* glycoprotein, *PCI* percutaneous coronary intervention, *RCA* right coronary artery, *SS* syntax score

### Association of EFT with different outcomes

Studying the relation of echo-measured EFT with other possible determinants of CAD (Table 4), regression analysis demonstrated that EFT was significantly associated with increased BMI, fat mass, and LDL. Moreover, it was also significantly associated with higher odds of aorto-ostial lesions, LM disease, moderate/high-SS, high thrombus burden, no-reflow, and 6-month MACE.

**Table 4:** Predictability of epicardial fat thickness for different parameters

Logistic regression analysis for predictability of EFT for different categorical dependent variables			
	OR	95% CI	*P-*value
In-hospital MACE	1.06	0.78–1.44	0.68
6-month MACE	1.83	1.32–2.52	< 0.001
Aorto-ostial lesions	1.90	1.13–3.17	0.01
Left-main disease	2.59	1.60–4.19	< 0.001
Proximal lesions	0.83	0.67–1.02	0.08
High thrombus burden (G4-5)	1.80	1.38–2.34	< 0.001
GP-IIb/IIIa inhibitors	1.33	1.06–1.67	0.01
No-reflow (TIMI-flow ≤ 2)	1.73	1.25–2.40	0.001
Moderate/high syntax score	8.46	4.24–16.88	< 0.001

Linear regression analysis for predictability of EFT for different continuous dependent variables			
	Beta	95% CI	*P-*value
BMI	1.52	1.17–1.86	< 0.001
Fat mass (Kg)	3.25	2.20–4.31	< 0.001
LDL (mg/dl)	12.17	7.57–16.78	< 0.001
TGL/HDL Ratio	0.41	0.11–0.70	0.007
Syntax score	4.28	3.66–4.90	< 0.001
Grace score	11.25	9.33–13.18	< 0.001

*BMI* body mass index, *CI* confidence interval, *EFT* epicardial fat thickness, *MACE* major adverse cardiovascular events, *OR* Odds ratio

**Fig. 2:**
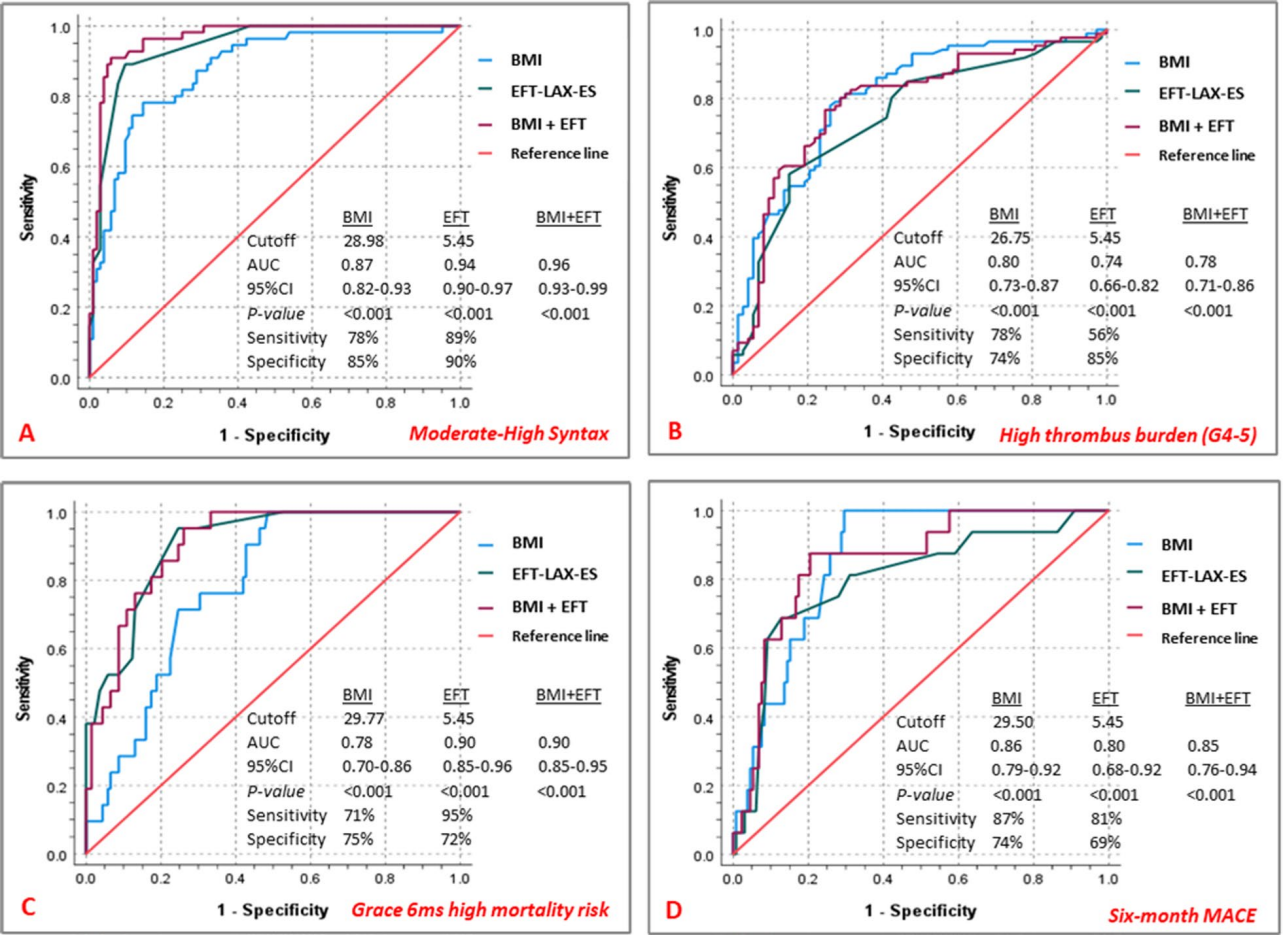
Receiver-operating characteristic (ROC) analysis for the predictability of body mass index (BMI), epicardial fat thickness (EFT), and combined BMI and EFT for moderate/high syntax (**A**), high thrombus burden (**B**)*,* Grace 6-months high mortality risk (**C**), and six-month MACE (**D**). *AUC* area under the curve, *BMI* body mass index, *CI* confidence interval, *EFT* epicardial fat thickness, *MACE* major adverse cardiovascular events

**Fig. 3:**
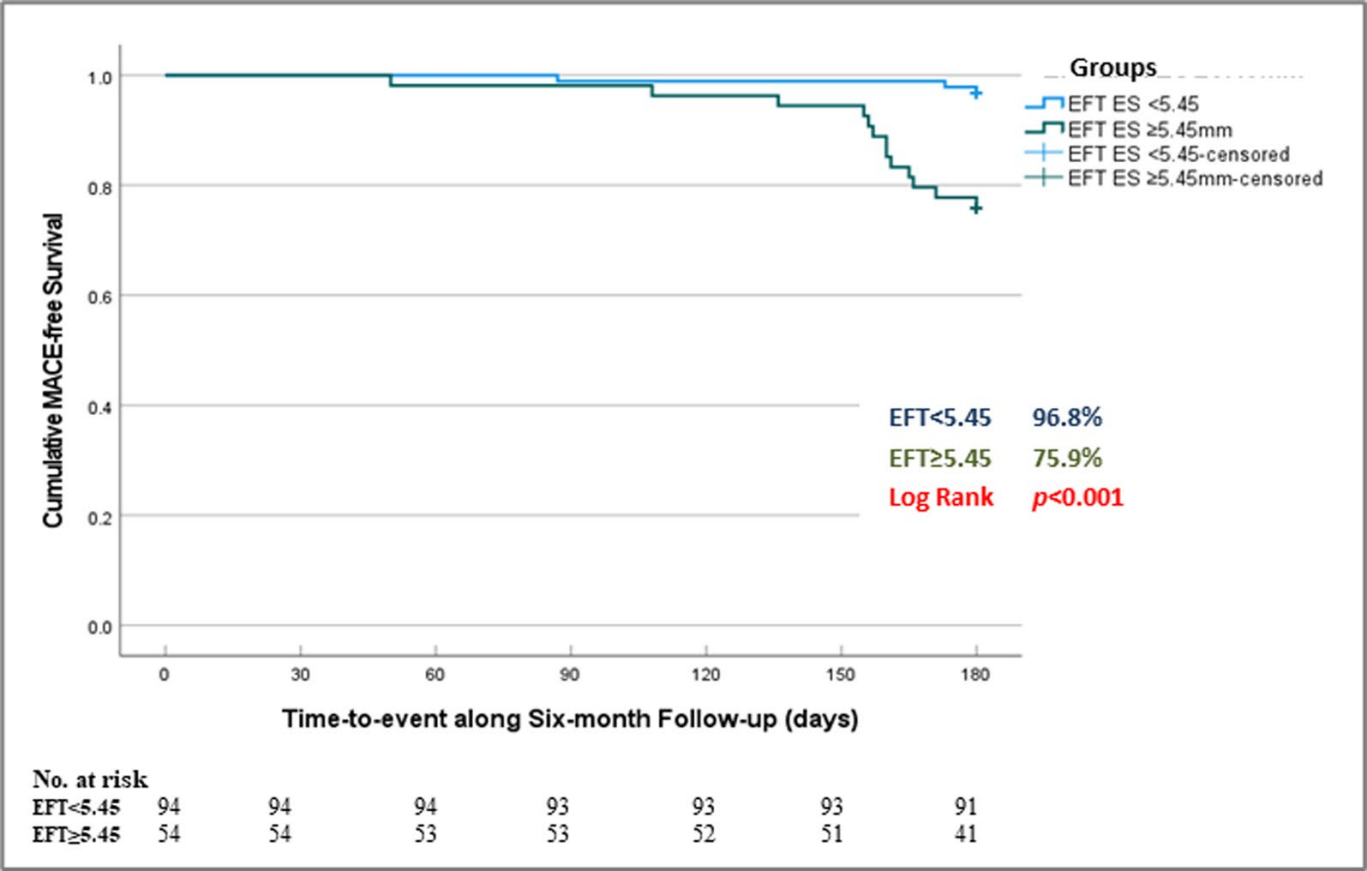
Kaplan–Meier curves for cumulative MACE-free survival among patient groups based on epicardial fat thickness cutoff value ≥ 5.45 mm. EFT: epicardial fat thickness, *ES* end-systole

ROC-analysis (Fig. 2) demonstrated that the generated EFT cutoff value of 5.45 mm was strongly predictive of moderate/high-SS (AUC = 0.94, 95%CI 0.90–0.97, sensitivity = 89%, specificity = 90%, and *p* < 0.001). Using the same cutoff (5.45 mm), EFT showed significantly strong predictability for high thrombus burden (AUC = 0.74, 95% CI 0.66–0.82, sensitivity = 56%, specificity = 85%, and *p* < 0.001), Grace 6-month high mortality risk (AUC = 0.90, 95% CI 0.85–0.96, sensitivity = 95%, specificity = 72%, and *p* < 0.001), as well as 6-month MACE (AUC = 0.80, 95% CI 0.68–0.92, sensitivity = 81%, specificity = 69%, and* p* < 0.001). Based on the ROC-derived cutoff value for EFT, survival analysis showed significantly higher 6-month MACE rate among the group with EFT ≥ 5.45 mm compared to those < 5.45 mm (log rank *p* < 0.001) (Fig. 3).

Addressing possible collinearity between BMI and EFT, our results demonstrated significant moderate correlation between EFT and BMI (r = 0.57, p < 0.001), and significant association of higher BMI with increased EFT (Beta = 1.52, 95% CI 1.17–1.86, *p* < 0.001). Hence the individual predictive capacity of each, as well as their complementary impact on the study outcomes were assessed. In this respect, our results showed that the predictability of combined BMI and EFT was (AUC = 0.96, 95% CI 0.93–0.99, *p* < 0.001) for severe CAD (moderate/high SS), (AUC = 0.78, 95% CI 0.71–0.86, *p* < 0.001) for high coronary thrombus burden (grades 4 and 5), (AUC = 0.90, 95% CI 0.85–0.95, *p* < 0.001) for Grace 6-month high mortality risk, and (AUC = 0.85, 95% CI 0.76–0.94, *p* < 0.001) for 6-month MACE (Fig. 2). 

Moreover, multivariable regression analysis (Table 5) showed that EFT (OR = 7.38, 95% CI 2.99–18.16, *p* < 0.001) and BMI (OR = 1.65, 95% CI 1.25–2.19, *p* < 0.001) were significant independent predictors for severe CAD (moderate/high-SS). EFT was also a significant independent predictor of the Grace 6-ms high mortality risk (OR = 2.36, 95%CI 1.25–4.47, *p* = 0.008), along with age (OR = 1.09, 95% CI 1.01–1.19, *p* = 0.02).

**Table 5:** Predictors of severe CAD (Moderate/high SS) and Grace 6-months high mortality risk

Variables	Univariable analysis			Multivariable analysis		
	OR	95% CI	*P-*value	OR	95% CI	*P-*value
Logistic regression analysis for predictors of moderate/high syntax score						
Age	1.03	1.00–1.06	0.04	1.06	0.99–1.13	0.08
Male gender	0.91	0.35–2.33	0.85			
Waist circumference (cm)	1.08	1.04–1.12	< 0.001			
Hip circumference (cm)	1.08	1.04–1.12	< 0.001			
BMI (kg/m^2^)	1.59	1.37–1.85	< 0.001	1.65	1.25–2.19	< 0.001
Fat mass% (Navy)	1.08	1.04–1.12	< 0.001	0.95	0.88–1.04	0.40
LDL-C (mg/dl)	1.01	1.01–1.03	< 0.001	0.99	0.98–1.01	0.70
Triglycerides/HDL ratio	1.24	1.07–1.43	0.004	1.09	0.82–1.46	0.53
EFT-ES PLAX (mm) *	8.46	4.24–16.88	< 0.001	7.38	2.99–18.16	< 0.001
Logistic regression analysis for predictors of Grace 6 months high mortality risk						
Age	1.04	1.00–1.09	0.02	1.09	1.01–1.19	0.02
Male gender	0.63	0.19–2.10	0.46			
Smoking	3.97	1.11–14.14	0.03	1.13	0.14–9.13	0.90
Diabetes mellitus	1.41	0.53–3.78	0.48			
BMI (kg/m^2^)	1.22	1.09–1.35	< 0.001	0.92	0.76–1.11	0.39
Triglycerides/HDL ratio	1.10	0.96–1.26	0.14			
Anterior STEMI	1.35	0.46–3.93	0.58			
LV ejection fraction (%)	0.97	0.91–1.02	0.30			
EFT-ES PLAX (mm) **	3.31	2.08–5.26	< 0.001	2.36	1.25–4.47	0.008
Proximal lesions	2.76	0.96–7.97	0.05			
High thrombus burden (G4-5)	10.06	2.25–44.88	0.002	7.09	0.59–83.92	0.12
No-reflow (TIMI-flow ≤ 2)	11.00	3.24–37.32	< 0.001	4.25	0.71–25.16	0.11
Syntax score	1.25	1.13–1.39	< 0.001	1.16	0.99–1.35	0.06

*BMI* body mass index, *CI* confidence interval, *ES* end-systole, *EFT* epicardial fat thickness, *HDL* high density lipoprotein, *LDL* low density lipoprotein, *LV* left ventricle, *MACE* major adverse cardiovascular events, *OR* Odds ratio, *PLAX* parasternal long-axis, *STEMI* ST-elevation myocardial infarction

^*^Corrected for age, BMI, fat mass% (navy), LDL, and triglycerides/HDL ratio

^**^Corrected for age, smoking, BMI, high thrombus burden, no-reflow, and the syntax score

## Discussion

The main findings of our study demonstrated that echo-measured EFT was significantly larger among STEMI patients having moderate/high syntax scores. Our study proposed a single ROC-derived EFT cutoff value of ≥ 5.45 mm that significantly and consistently discriminated moderate/high-SS, high coronary thrombus burden, Grace 6-month high mortality risk, and 6-month MACE among STEMI patients with reasonable respective sensitivity and specificity. Moreover, EFT was among the independent predictors of severe CAD (moderate/high-SS) and 6-month mortality risk on multivariable analysis.

### Relation between markers of adiposity, epicardial fat, and CAD

An intriguing observation from our study was the association between anthropometric measurements of adiposity (higher BMI and fat mass) and increased EFT and PFT among STEMI patients having moderate/high SS. This was in line with previous findings that adiposity indices were significantly correlated to and predicted high syntax [11]. The moderate/high SS group of our study also had significantly higher total cholesterol, triglycerides, LDL, and high AIP, which was previously demonstrated to be a marker of CAD [6].

Moreover, our results showed a significant association of increased EFT with higher BMI, body fat mass, and LDL cholesterol, respectively. Despite of evident collinearity between BMI and EFT, yet our study showed reasonable predictability of each of them individually as well as of both in a complementary prediction model for moderate/high SS as demonstrated on both ROC-analysis and multivariable regression analysis. These findings collectively highlight the intricate interplay between adiposity, metabolic factors, and atherosclerosis. However, our results showed that compared to BMI, EFT had higher predictability for moderate-high SS (AUC = 0.94 vs 0.87) and for 6-months high mortality risk (AUC = 0.90 vs 0.78) with higher odds of severe CAD than BMI (OR = 7.38 vs 1.65).

### Epicardial fat and its relation to acute coronary syndromes

Incorporating EFT in risk assessment for CAD could potentially guide primary and secondary preventive strategies among STEMI patients. It was previously demonstrated that increased epicardial fat volume measured on computed tomography (CT) was significantly associated with increased risk of incident MI, fatal and non-fatal cardiac events among 4093 participants at 8 years, independent of traditional risk factors and coronary calcium score [12].

Earlier studies demonstrated an association of EAT and peri-coronary adipose tissue-induced vascular inflammation with the development and progression of CAD, coronary plaque calcification [13], plaque vulnerability [14, 15], and plaque rupture [16], hence inducing ACS. It was previously demonstrated that patients with MI have higher peri-coronary CT attenuation, a marker of increased inflammation, compared to those with stable CAD [17, 18], and that larger epicardial fat volume and higher attenuation were independently associated with the development of MI [19].

Noticeably, our results demonstrated that higher EFT was significantly associated with higher odds of heavy coronary thrombus burden (OR = 1.80, 95% CI 1.38–2.34, *p* < 0.001) and no-reflow (TIMI flow ≤ 2) (OR = 1.73, 95% CI 1.25–2.40, *p* < 0.001) among STEMI patients. Moreover, our results showed that the same cutoff value of EFT ≥ 5.45 mm was significantly associated with increased severity of CAD and similarly with high coronary thrombus burden with reasonable respective sensitivity and specificity. It is possible that the high inflammatory state in association with high-risk plaques among these patients has induced more aggressive thrombosis that contributed to higher odds of no-reflow.

### Predictability of epicardial fat thickness for severity of CAD

The impact of increased EFT goes beyond its mere association with the presence of atherosclerosis and acute coronary events and extends to impact the severity and complexity of the underlying CAD. Our results showed a significant strong correlation between EFT and the syntax score, which is an established tool defining CAD severity and complexity. This was in line with lately published data that demonstrated a strong positive correlation (r = 0.93) of echo-measured EFT with the SS among 324 CAD patients [20], yet including patients with chronic stable angina and ACS in contrary to our study that was exclusively conducted on STEMI patients.

Moreover, the results demonstrated that our ROC-generated EFT cutoff value ≥ 5.45 mm significantly predicted moderate/high-SS (SS > 22) with 89% sensitivity and 90% specificity. Previous studies proposed various cutoffs for EFT in the range of (4.65–5.5 mm) as predictors for the presence of significant CAD [21–25]. The variability of the cutoff values among these studies might be attributed to the lack of a defined standardized approach for measuring EFT and their variability in defining CAD severity. Additionally, ethnic differences might also explain the presence of various cutoffs in relation to CAD severity. In an earlier study, EFT ≥ 4.7 mm significantly predicted high SS (≥ 33) among 373 Chinese STEMI patients and increased EFT was an independent predictor of in-hospital MACE [26]. Another study showed that EFT was strongly correlated with the severity of CAD assessed by Gensini score (r = 0.91) and that EFT ≥ 4.75 mm was a predictor for the mere presence of significant CAD, whereas a higher cutoff ≥ 5.2 mm showed better predictability for two- and three-vessel disease [27].

On the other hand, a recent study emphasized indexing epicardial fat measurements and showed that echo-measured EFT ≥ 4.15 mm/m^2^ significantly predicted SS > 22, though limited by a small sample size. Moreover, it showed that larger both non-indexed and indexed-EFT were significantly correlated with increased epicardial adipose tissue oxidative stress, and that indexed-EFT significantly predicted increased inflammation [28]. This underscores the active role of epicardial fat in the pathogenesis of coronary atherosclerosis rather than being a mere determinant of visceral adiposity.

### Impact of epicardial adipose tissue on clinical outcomes among STEMI patients

Our results demonstrated that increased EFT did not impact the rate of in-hospital MACE (OR = 1.06, 95% CI 0.78–1.44, *p* = 0.68). In contrary, Wang et al. demonstrated that echo-measured EFT was a significant independent predictor for in-hospital MACE among patients with acute STEMI [26]. Notably, our study confined the definition of in-hospital MACE to the composite of re-infarction, heart failure, and fatal arrhythmia, excluding those who did not survive the in-hospital duration. This might have limited our conclusions, yet it was based on the concern that inclusion of in-hospital deaths reflects underlying high-risk clinical condition rather than an association with increased EFT.

Regarding follow-up MACE, our results demonstrated significantly higher MACE among those with higher EFT(≥ 5.45 mm). It was previously demonstrated that echo-measured EFT significantly predicted long-term MACE at 3-year follow-up among STEMI patients [29]. Another study demonstrated that echo-measured EFT showed higher predictability for MACE than BMI, LV EF, and the syntax score among patients with CAD [30]. Systematic analysis of nine studies (2306 patients), of which 2136 had echo-measured EFT and 1527 STEMI patients, demonstrated a negative impact of epicardial fat on clinical outcomes and the estimated risk of future adverse events [31].

On the other hand, our study evaluated both the in-hospital and 6-months mortality risks using the Grace score, demonstrating that increased EFT was significantly associated with increased Grace score (Beta = 11.25, 95% CI 9.33–13.18, p < 0.001), and was among the independent predictors of Grace score-derived 6-month high mortality risk. Interestingly, our study demonstrated that applying the same EFT cutoff ≥ 5.45 mm that significantly predicted CAD severity and high coronary thrombus burden among our STEMI patients effectively predicted 6-month high mortality risk (AUC = 0.90, 95% CI 0.85–0.96, sensitivity = 95%, specificity = 72%, and *p* < 0.001). Previous studies demonstrated a significant correlation between echo-measured EFT and clinical Grace and TIMI risk scores among ACS patients [32, 33], however did not propose respective EFT cutoff values. Noticeably, our study demonstrated no mortality at 6 months. This might be attributed to the relatively small sample size and strict inclusion criteria. A longer follow-up study adequately powered for mortality outcome is warranted.

### Reliability of echo-measured epicardial fat thickness

Echocardiography provides a low-cost, easily accessible, and simple tool for the assessment of EFT in daily clinical practice with reasonable reproducibility. Yet, so far, there is no consensus about a standard echocardiographic method for the measurement of EFT in terms of the view used, the exact location, and the optimal timing of measurement (end-systolic frames allow better delineation of EFT rather than end-diastolic frames where it gets compressed). In this respect, our study presented a detailed echocardiographic assessment of EFT using the different proposed measurement methods, showing minimal variations. We demonstrated that EFT was readily measurable for all patients on the PLAX view, while 7.5% had unmeasurable EFT on the short-axis view.

Notably, our echo-measured EFT had an excellent intra- and inter-observer agreement and showed a significant moderate correlation with corresponding CMR-measured fat areas. Nevertheless, a stronger correlation with CMR data might have been evident using large CMR-datasets and comparing corresponding echo and CMR-measured fat thickness rather than area. Iacobellis et al. demonstrated an excellent correlation between PLAX echo-measured EFT at end-systole and that measured on CMR using corresponding points and views on the RV-free wall and suggested a regression equation for the corresponding CMR-derived epicardial fat area [4]. Hence, emphasizing the feasibility and validity of echo-measured EFT particularly from the PLAX view at end-systole. Based on this, our study proposed an ROC-generated single EFT cutoff value of ≥ 5.45 mm that was readily derived from average measurements applied on the PLAX view at end-systole, and that demonstrated significant and consistent predictability for multiple outcome points, namely severe and complex CAD, high coronary thrombus burden, high 6-month mortality risk, and 6-month MACE.

To summarize, echo-measured EFT might represent a simple risk marker and a possible therapeutic target with prognostic implications among STEMI patients. Large-scale studies are needed to address these issues. Moreover, adopting standardized protocols for echo-measured epicardial fat thickness would enhance consistency and reliability across studies. We assume that our findings pave the way for future studies validating our predictive EFT cutoff value and incorporating it with other established risk models and therapeutic targets, for better management of STEMI patients.

### Limitations

The relatively small size and cross-sectional study design were possible limitations. Unfortunately, it is not routine at our institute to have CMR studies for all STEMI patients during their in-hospital stay or shortly thereafter due to logistic and financial constraints. The small subset of included CMR patients was confined to those who had their CMR study performed within 30 days from the incident MI. Excluding patients who had hemodynamic instability on admission as well as the in-hospital deaths might have induced potential selection bias, limiting our conclusions to relatively stable patients with initially lower in-hospital mortality risk. An extended follow-up of MACE events might have better reflected the impact of EFT on the mortality outcomes.

## Conclusions

Echo-measured EFT showed a significant association with increased severity of CAD among STEMI patients. A single ROC-derived cutoff value for EFT of ≥ 5.45 mm significantly predicted the outcomes of severe CAD, high coronary thrombus burden, Grace score 6-month mortality risk, and 6-momths MACE. Increased EFT was an independent predictor of moderate-high SS and 6-month mortality risk. 

## Supplementary Information

Supplementary material 1: Table S1. CMR data of the study population (n=43). Table S2. Correlations between Echo-derived EFT and PFT and corresponding CMR-areas. Table S3. Comparative analysis between the syntax-score categories. Figure S1. Flow-chart of the study population. BMI: body mass index, EFT: epicardial fat thickness, ES: end-systole, MACE: major adverse cardiovascular events, MI: myocardial infarction PLAX: parasternal long-axis, PPCI: primary percutaneous coronary intervention, STEMI: ST-elevation myocardial infarction, TLR: target lesion revascularization. Figure S2. Examples of echo-measured epicardial and pericardial fat thickness (upper panel) and corresponding CMR-measured fat areas (lower panel). (A): parasternal long-axis view end-systolic frame, (B): parasternal short-axis view end-systolic frame, (C): epicardial fat area, (D): pericardial fat area, (1): epicardial fat thickness, and (2): pericardial fat thickness

## Data Availability

No datasets were generated or analysed during the current study.

## Declarations

### **Clinical trial registration**

This study was registered at ClinicalTrials.gov ID: (NCT03308539), October 2017.
