# Supplementary material for: Epicardial fat thickness predicts severe coronary artery disease and high mortality risk among ST-elevation myocardial infarction patients
Source: Echo Res Pract. 2025 Jul 21;12:17. doi: 10.1186/s44156-025-00087-y (PMC12278483; doi:10.1186/s44156-025-00087-y)
Supplement: Supplementary file 1 — Supplementary material 1: Table S1. CMR data of the study population (n=43). Table S2. Correlations between Echo-derived EFT and PFT and corresponding CMR-areas. Table S3. Comparative analysis between the syntax-score categories. Figure S1. Flow-chart of the study population. BMI: body mass index, EFT: epicardial fat thickness, ES: end-systole, MACE: major adverse cardiovascular events, MI: myocardial infarction PLAX: parasternal long-axis, PPCI: primary percutaneous coronary intervention, STEMI: ST-elevation myocardial infarction, TLR: target lesion revascularization. Figure S2. Examples of echo-measured epicardial and pericardial fat thickness (upper panel) and corresponding CMR-measured fat areas (lower panel). (A): parasternal long-axis view end-systolic frame, (B): parasternal short-axis view end-systolic frame, (C): epicardial fat area, (D): pericardial fat area, (1): epicardial fat thickness, and (2): pericardial fat thickness [file 44156_2025_87_MOESM1_ESM.docx]

**Table S1. CMR data of the study population (n=43)**

|  | **All CMR-pts**  **(n=43)** | **Low SS**  **(n=26) (60.5%)** | **Moderate/High SS**  **(n=17) (39.5%)** | ***P-*value** |
| --- | --- | --- | --- | --- |
| **STEMI-CMR** (days) | 19.93±8.22  21(14-27) | 18.31±8.23  18(12.7-24.5) | 22.41±7.79  22(19.5-28.0) | 0.11  0.07 |
| **LV EDV** (ml) | 157.23±33.07 | 161.62±31.06 | 150.51±35.84 | 0.28 |
| **LV ESV** (ml) | 86.31±28.95 | 88.15±28.73 | 83.50±29.94 | 0.61 |
| **LV EF** (%) | 45.85±10.75 | 46.15±10.61 | 45.40±11.26 | 0.82 |
| **LV Stroke volume** (ml) | 71.53±18.26 | 73.80±17.49 | 68.05±19.39 | 0.31 |
| **LV Stroke index** | 39.97±9.25 | 41.57±9.05 | 37.52±9.29 | 0.16 |
| **LV wall mass** | 95.90±26.83 | 94.47±26.50 | 98.10±28.00 | 0.67 |
| **LV wall/papillary mass** | 133.79±30.51 | 133.54±28.96 | 134.18±33.66 | 0.94 |
| **RV EDV** (ml) | 113.41±29.89 | 114.11±28.54 | 112.34±32.73 | 0.85 |
| **RV ESV** (ml) | 46.64±17.46 | 46.06±16.34 | 47.52±19.55 | 0.79 |
| **RV EF** (%) | 58.70±10.06 | 59.21±9.77 | 57.91±10.75 | 0.68 |
| **RV Stroke volume** (ml) | 67.42±20.28 | 68.06±20.42 | 66.42±20.67 | 0.79 |
| **RV Stroke index** | 37.24±10.45 | 37.90±10.77 | 36.39±10.30 | 0.66 |
| **Epicardial-fat area** (cm^2^) | 932.9±521.9  906 (674-1121) | 837.3±404.3  837 (648-1087) | 1079.1±649.8  1079 (738-1271) | 0.14  0.47 |
| **Pericardial-fat area** (cm^2^) | 1145.9±787.8  998 (636-1388) | 898.1±449.5  898 (572-1266) | 1524.9±1030.1  1524 (819-2019) | **0.009**  **0.01** |

**CMR:** cardiac magnetic resonance, **EDV:** end-diastolic volume, **EF:** ejection fraction, **ESV:** end-systolic volume, **LV**: left ventricle, RV**:** right ventricle, **SS:** syntax score, **STEMI:** ST-elevation myocardial infarction.

**Table S2. Correlations between Echo-derived EFT and PFT and corresponding CMR- areas**

| **Variables /**  ***r (p-value)*** | **CMR**  **Epi-area-ED** | **Variables /**  ***r (p-value)*** | **CMR**  **Peri-area-ED** |
| --- | --- | --- | --- |
| **EFT PLAX-ES** | 0.36 (p=0.01) | **PFT PLAX-ES** | 0.44 (p=0.008) |
| **EFT PLAX-ED** | 0.35 (p=0.02) | **PFT PLAX-ED** | 0.55 (p=0.001) |
| **EFT PSAX-ES** | 0.39 (p=0.01) | **PFT PSAX-ES** | 0.46 (p=0.006) |
| **EFT PSAX-ED** | 0.39 (p=0.01) | **PFT PSAX-ED** | 0.58 (p<0.001) |

**CMR:** cardiac magnetic resonance, **ED:** end-diastole, **EFT:** epicardial fat thickness, **ES:** end-systole, **PFT:** pericardial fat thickness, **PLAX:** parasternal long-axis, **PSAX:** parasternal short-axis.

**Table S3. Comparative analysis between the syntax-score categories**

|  | **Low SS**  **(n=104) (65.4%)** | **Moderate SS**  **(n=45) (28.3%)** | **High SS**  **(n=10) (6.3%)** | ***P-*value** |
| --- | --- | --- | --- | --- |
| **Age** (years) | 54.69±12.74 | 59.33±10.52 | 56.20±10.52 | 0.09 |
| **Male Gender** | 90(86.5%) | 39(86.7%) | 8(80.0%) | 0.84 |
| **Smoking** | 52(50.0%) | 40(88.9%) | 9(90.0%) | **<0.001** |
| **Hypertension** | 20(19.2%) | 14(31.1%) | 3(30.0%) | 0.25 |
| **Diabetes mellitus** | 27(26.0%) | 14(31.1%) | 2(20.0%) | 0.70 |
| **Waist circumference** (cm) | 122.51±15.39 | 134.33±10.77 | 140.70±11.93 | **<0.001 (A,C)** |
| **Hip circumference** (cm) | 120.35±14.97 | 131.42±10.48 | 137.20±10.71 | **<0.001(A,C)** |
| **BSA** (m^2^) | 1.82±0.13 | 1.92±0.14 | 1.94±0.15 | **<0.001 (A,C)** |
| **BMI** (kg/m^2^) | 26.23±2.90 | 31.08±3.95 | 34.53±4.86 | **<0.001 (A,B,C)** |
| **Fat mass** (kg) | 27.81±10.14 | 37.86±11.46 | 43.36±15.18 | **<0.001 (A,C)** |
| **Lipid profile**  Total cholesterol (mg/dl)  LDL-C (mg/dl)  HDL-C (mg/dl)  Triglycerides (mg/dl)  Atherogenic plasma index | 205.88±50.06  125.45±41.67  41.13±8.99  154.34±75.91  0.54±0.22 | 241.81±60.43  162.03±48.28  38.64±7.85  197.29±98.00  0.66±0.23 | 286.60±77.99  179.90±74.46  36.75±10.76  248.00±116.79  0.79±0.27 | **<0.001 (A,C)**  **<0.001 (A,C)**  0.14  **0.001 (A,C)**  **0.001 (A,C)** |
| **Anterior STEMI** | 73(70.2%) | 36(80.0%) | 4(40.0%) | **0.03** |
| **Total ischemic time** (hrs) | 5.16±1.93 | 4.80±1.71 | 4.90±1.79 | 0.53 |
| **Peak-CK** | 1302(894-2087) | 1587(1087-2668) | 1598(1098-2360) | 0.18 |
| **Complete ST-resolution** | 99(95.2%) | 43(95.6%) | 7(70.0%) | **0.006** |
| **In-hospital MACE** | 9(8.7%) | 7(15.6%) | 1(10.0%) | 0.45 |
| **Grace score** | 87.43±15.37 | 124.71±15.62 | 154.40±28.09 | **<0.001 (A,B,C)** |
| **LV EF** (%) | 47.85±8.89 | 45.36±8.51 | 46.50±5.14 | 0.26 |
| **EFT-ES PLAX** (n=159) | 4.34±1.01 | 6.43±1.13 | 8.20±1.37 | **<0.001 (A,B,C)** |
| **EFT-ES PSAX** (n=147) | 4.44±0.95 | 6.58±1.12 | 8.64±1.29 | **<0.001 (A,B,C)** |
| **PFT-ES PLAX** (n=142) | 4.48±1.17 | 6.46±1.06 | 7.47±1.22 | **<0.001 (A,C)** |
| **PFT-ES PSAX** (n=115) | 4.48±1.09 | 6.40±1.03 | 7.33±0.81 | **<0.001 (A,C)** |
| **Aorto-ostial lesions** | 0(0.0%) | 3(6.7%) | 1(10.0%) | **0.01** |
| **Left-main disease** | 0(0.0%) | 5(11.1%) | 3(30.0%) | **<0.001** |
| **Three-vessel disease** | 8(7.7%) | 14(31.1%) | 5(50.0%) | **<0.001** |
| **Proximal lesions** | 50(48.1%) | 34(75.6%) | 6(60.0%) | **0.008** |
| **High thrombus burden** | 35(33.7%) | 43(95.6%) | 8(80.0%) | **<0.001** |
| **No-reflow** (TIMI-flow≤2) | 3(2.9%) | 6(13.3%) | 4(40.0%) | **<0.001** |
| **Syntax score** | 14.55±5.24 | 25.60±2.82 | 42.60±12.17 | **<0.001 (A,B,C)** |

**BMI:** body mass index, **BSA:** body surface area, **EFT:** epicardial fat thickness, **HDL**: high density lipoprotein, **LDL**: low density lipoprotein, **MACE:** major adverse cardiovascular events, **PFT:** pericardial fat thickness, **SS:** syntax score, **STEMI:** ST-elevation myocardial infarction.

***p*-value: denotes level of significance between groups. Post-Hoc Bonferroni within groups significance was denoted by (A) between low and moderate syntax, (B) between moderate and high syntax, and (C) between high and low syntax, respectively.**


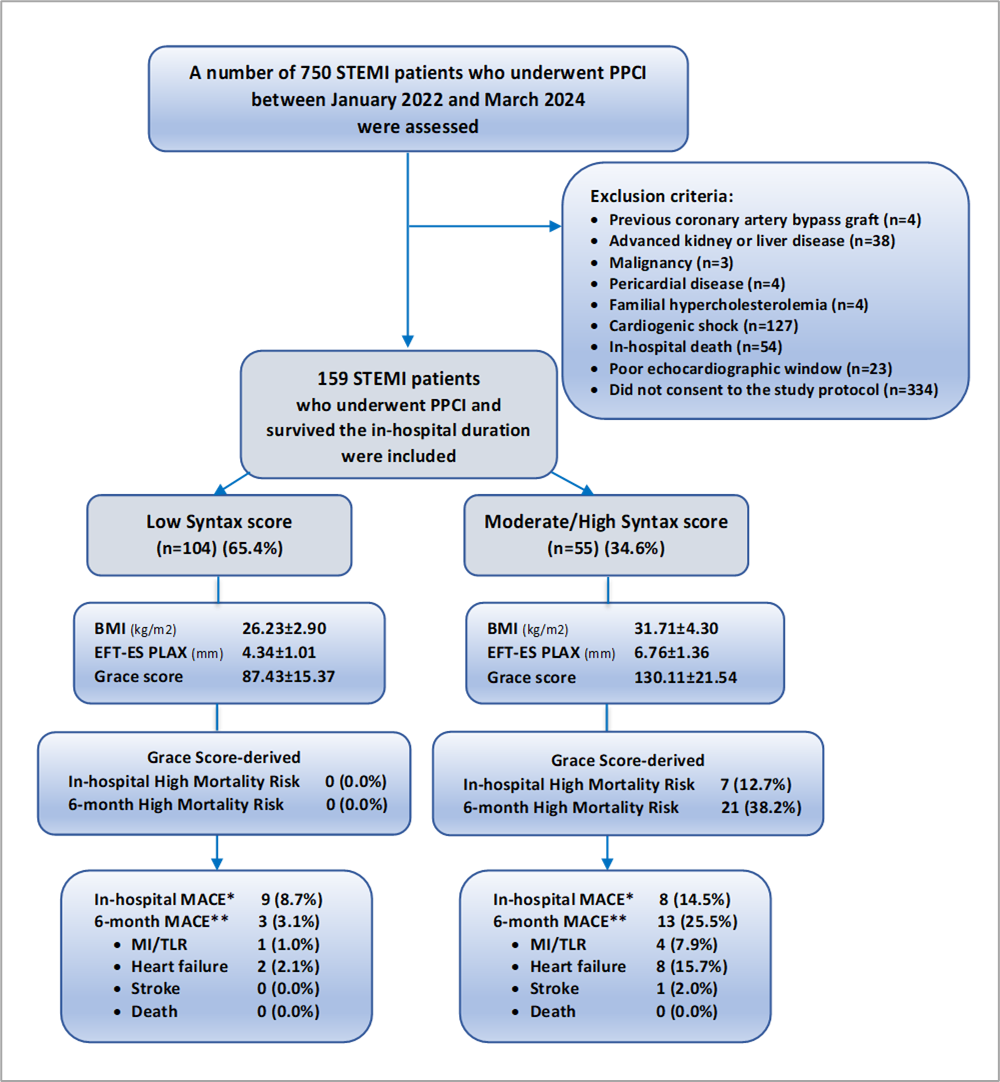


**Figure S1. Flow-chart of the study population. BMI:** body mass index, **EFT:** epicardial fat thickness, **ES:** end-systole, **MACE:** major adverse cardiovascular events, **MI:** myocardial infarction **PLAX:** parasternal long-axis, **PPCI:** primary percutaneous coronary intervention, **STEMI:** ST-elevation myocardial infarction, **TLR:** target lesion revascularization.

**Figure S2. Examples of echo-measured epicardial and pericardial fat thickness** *(upper panel)* **and CMR-measured epicardial and pericardial fat areas** *(lower panel)***. (A)**: parasternal long-axis view end-systolic frame, **(B)**: parasternal short-axis view end-systolic frame, **(C)**: epicardial fat area, **(D)**: pericardial fat area, **(1)**: epicardial fat thickness, and **(2)**: pericardial fat thickness.
